# Determining resuscitation threshold for extremely preterm infants based on the survival rates without severe neurological injury

**DOI:** 10.7189/jogh.13.04059

**Published:** 2023-05-26

**Authors:** Xiao-yu Dong, Wen-wen Zhang, Jun-ming Han, Dan Bi, Zhen-ying Yang, Xiao-liang Wang, Hui Wang, De-Juan Yang, Chun-lei Zhang, Rui Gao, Bing-jin Zhang, Li-li Hu, Simmy Reddy, Sen-kang Yuan, Yong-hui Yu

**Affiliations:** 1 Shandong Provincial Maternal and Child Health Care Hospital Affiliated to Qingdao University, Jinan, China; 2 Department of Paediatrics, Jinan Maternal and Child Health Hospital, Jinan, China; 3 Department of Paediatrics, Shandong Provincial Hospital Affiliated to Shandong First Medical University, Jinan, China; 4 Department of Paediatrics, Qilu Hospital Affiliated to Shandong University, Jinan, China; 5 Department of Paediatrics, Taian Maternal and Child health Care Hospital, Taian, China; 6 Department of Paediatrics, Yantai Yuhuangding Hospital, Yantai, China; 7 Department of Paediatrics, Hebei PetroChina Central Hospital, Langfang, China; 8 Department of Paediatrics, The First Affiliated Hospital of Shandong First Medical University, Jinan China; 9 Department of Paediatrics, Wei Fang Maternal and Child Health Hospital, Weifang, China; 10 Department of Paediatrics, Liaocheng People's Hospital, Liaocheng, China; 11 Department of Paediatrics, Shengli Oilfield Central Hospital, Dongying, China; 12 Department of Paediatrics, Baogang Third Hospital of Hongci Group, Baotou, China; 13 Department of Paediatrics, Cheeloo College of Medicine, Shandong University, Jinan, China; 14 Inspur Electronic Information Industry Co. Ltd, China

## Abstract

**Background:**

Published guidelines on decision-making and resuscitation of extremely preterm infants primarily focus on high-income countries. For rapidly industrializing ones like China, there is a lack of population-based data for informing prenatal management and practice guidelines.

**Methods:**

The Sino-northern Neonatal Network conducted a prospective multi-centre cohort study between 1 January 2018 and 31 December 2021. Infants with a gestational age (GA) between 22 (postnatal age in days = 0) and 28 (postnatal age in days = 6) admitted to 40 tertiary NICUs in northern China were included and evaluated for death or severe neurological injury before discharge.

**Results:**

For all extremely preterm infants (n = 5838), the proportion of admission to the neonatal was 4.1% at 22-24 weeks, 27.2% at 25-26 weeks, and 75.2% at 27 and 28 weeks. Among 2228 infants admitted to the NICU, 216 (11.1%) were still elected for withdrawal of care (WIC) due to non-medical factors. Survival rates without severe neurological injury were 6.7% for infants at 22-23 weeks, 28.0% at 24 weeks, 56.7% at 24 weeks, 61.7% at 25 weeks, 79.9% at 26 weeks, and 84.5% at 27 and 28 weeks. Compared with traditional criterion at 28 weeks, the relative risk for death or severe neurological injury were 1.53 (95% confidence interval (CI) = 1.26-1.86) at 27 weeks, 2.32 (95% CI = 1.73-3.11) at 26 weeks, 3.62 (95% CI = 2.43-5.40) at 25 weeks, and 8.91 (95% CI = 4.69-16.96) at 24 weeks. The NICUs with higher proportion of WIC also had a higher rate of death or severe neurological injury after maximal intensive care (MIC).

**Conclusions:**

Compared to the traditional threshold of 28 weeks, more infants received MIC after 25 weeks, leading to significant increases in survival rates without severe neurological injury. Therefore, the resuscitation threshold should be gradually adjusted from 28 to 25 weeks based on reliable capacity.

**Registration:**

China Clinical Trials Registry. ID: ChiCTR1900025234.

Preterm birth and ensuing complications are the primary causes of death among children under five years of age globally [1]. Although extremely preterm infants account for only 5.2% of all preterm births, they receive significant attention due to their high mortality and sequelae rates [2]. With the development of assisted reproductive technologies and perinatal medical system and related life-saving technologies, the survival rates of preterm infants have dramatically improved [3,4]. Consequently, health care workers and policymakers always consider the survival and adverse neurological sequelae of this special group, leading to changes in the resuscitation threshold over time. In developed countries during the 1960s to 1970s, the resuscitation threshold included infants with a gestational age >28 weeks. However, there is general agreement for active care at 24-25 weeks of GA; in several countries, including Japan, the threshold is currently set at 22 completed weeks of gestation [5,6].

As the largest developing country, China has over 1.17 million premature infants, the second largest number of premature babies globally [2]. Previously, due to the limitation of care capacity in NICUs and the absence of up-to-date data, preterm births have been defined as deliveries with GA between 28 and 37 weeks [7]. More recently, with rapid industrialisation, China has made significant progress in neonatal treatment. From 1990 to 2019, the newborn mortality rate in China declined 3.6 times more than the global average, nearing that of North America and Europe [8]. Advances in neonatal medicine have also increased the survival rate of preterm infants before 28 weeks of gestation. Furthermore, the continuous population decline from 2016, with a birth rate of less than 1% in 2020 and 2021 [9], makes this group more “valuable” than before. Despite these improvements, due to the absence of up-to-date data and reliable comments, a recent single-centre study showed that for live births at 24-28 weeks, 72.2% did not receive active care in the delivery room [10]. Even among infants admitted to the NICU, the proportion of deaths due to withdrawal of care (WIC) resulting from economic factors exceeded half of the total deaths among extremely preterm infants in the NICU [11,12].

We prospectively defined the mode of death and analysed the results of the Care-Preterm Cohort study (2018-2021), a regional cohort of infants born at a gestational age between 22 (postnatal age in days = 0) and 28 (postnatal age in days = 6) weeks based on the Sino-northern Neonatal Network. We aimed to provide the updated mortalities and morbidities for each GA group in the real world to discuss the recovery threshold of extremely preterm infants.

## METHODS

### Study design and setting

The Care-Preterm study was based on the Sino-northern Neonatal Network (SNN), which collected data both prospectively and continuously. The Network was established to report on survival and outcomes in very preterm and very low birth weight infants born in this geographical region in order to conduct quality improvement. It is based in Shandong and covers a total of six provinces (Shandong, Shanxi, Shaanxi, Henan, Hebei, and Inner Mongolia) comprising 40 NICUs covering 29 general hospitals and 11 maternity hospitals (four provincial, 30 prefecture, and six county hospitals). Health insurance coverage is at more than 95% in the included provinces. Per the Maternal Health Handbook, pregnant women <32 weeks of gestation are intrauterine transferred to a tertiary maternal or general hospital equipped with a tertiary NICU. Newborns are transferred at the county, prefectural, and provincial levels on a tiered basis [13]. We implemented strict quality control referred by the Korean Neonatal Network [14], including three levels of quality controllers and site-visit monitoring to ensure the data quality. The chief quality controller conducted audits semi-annually to ensure quality data abstraction. We collected data electronically with a secure interface that protected confidentiality and privacy.

### Population

We included infants born at GA between 22 (postnatal age in days = 0) and 28 (postnatal age in days = 6) and admitted to participating NICUs between 1 January 2018 and 31 December 2021. We excluded infants who had known congenital anomalies or who were moribund (those in whom a decision was made at the time of birth not to provide resuscitative care).

### Definition of variables and outcomes

We determined GA by the best estimate based on early prenatal ultrasound examination, the last menstrual period, and the physical examination of infants at birth, in this order. We included the date of embryo transfer to determine GA for in vitro fertilization. Mortality was defined as death due to any cause prior to discharge from the NICU. We defined severe neurological injury (SNI) as intraventricular haemorrhage (IVH) (grade 3 or 4) and/or porencephalic ventricular leukomalacia (PVL) [15]. The standard practice in SNN for identifying cerebral lesions is to perform one or two cranial ultrasound scans during the first week after birth (generally in the first 48 hours) and then weekly for the following two weeks. For sites where cranial ultrasound cannot be performed, magnetic resonance imaging (MRI) is conducted at corrected GA of 36 weeks or at discharge.

We defined a composite outcome of mortality or any major morbidity as death before discharge or survival with any of four major morbidities: bronchopulmonary dysplasia (BPD), severe retinopathy of prematurity (ROP), SNI (IVH ≥grade 3 and/or PVL), stage 2 or 3 necrotic enterocolitis of newborn (NEC). We defined BPD as respiratory support given at 36 weeks’ post menstrual age or at discharge (if earlier than 36 weeks’ PMA) to level 2 centres [16], severe ROP as ROP stage 3, 4, 5, and/or those with ROP treatment (laser or intraocular injection) [17], severe NEC as stage 2 or 3, according to Bell ’definition [18]. Based on the actual therapy infants received, we divided the deaths into maximal intensive care (MIC) and WIC. MIC included life-sustaining therapies such as ventilatory and cardiovascular support and resuscitation efforts pursued until the infant was pronounced dead or when infants suffered severe neurological injury or when infants were in agonal situations. For redirected treatment, when these infants’ guardians may have signed a waiver, but chose redirection due to the infant being in a near-death state, we also categorised it as MIC for the death was due to rescue failure. Withdrawal of intensive care was defined as when the infant’s guardians requested termination of care for economic and social reasons, even though infants did not suffer severe neurological injury/were not in terminal status [19-21].

We defined normothermia as a recorded admission temperature between 36.5 and 37.5°C (rectal or axillary) taken within the first hour of admission and representing the first set of vital signs recorded after NICU admission [22].

### Statistical analysis

We summarised demographic and clinical characteristics using counts and percentages for categorical and medians and interquartile ranges (IQRs) for continuous variables. We used the χ^2^ or Fisher exact test to compare between groups. For internal comparison, we divided units with more than 20 cases into four groups, according to the proportion of WIC according to quartiles, descriptively compared the incidence of mortality or SNI after MIC after standardisation of potential confounders including male gender, antenatal corticosteroids, the Score for Neonatal Acute Physiology with Perinatal Extension II (SNAPPE-II) >20, and multiple births. We evaluated the associations between GA and mortality or SNI on a continuous scale, with restricted cubic spline curves based on logistic models with three knots at the 25th, 50th, and 75th percentiles of the distribution. We selected covariables that could be potential confounders of the associations between GA and infant outcome based on literature [23,24] and clinical knowledge, potential confounders, including male gender, antenatal corticosteroids, SNAPPE-II>20 and multiple births. We considered an alpha level >0.05 as statistically significant (all tests were two sided).

## RESULTS

### Study population

In the 40 hospitals included in the SNN, 5838 pregnancies at 22 (postnatal age in days = 0) and 28 (postnatal age in days = 6) weeks resulted in 2579 live births (Table S1 in the **Online Supplementary Document**). For all infants, the proportion of live births increased with GA from 5.2% at 22-23 weeks to 85.0% at 28 weeks. For all live births (n = 2579), 2228 infants (86.4%) were admitted to the NICUs; the proportion of live births by GA was 29.1% at 22-23 weeks, 46.5% at 24 weeks, 63.6% and 25 weeks, 70.3% at 26 weeks, 93.4% at 27 weeks, and 98.4% at 28 weeks, respectively. Non-invasive and invasive assisted ventilation equipment and pulmonary surfactants were supplied to each level of NICU, all provincial NICUs, 24 out of 30 prefectural and municipal hospitals, and 4 out of 6 county hospitals are capable of paediatric surgical consultation and surgery (Table S2 in the **Online Supplementary Document**).

### Comparison of characteristics between WIC and MIC

For 2228 extremely preterm infants, 1185 (56.6%) were male (median (md) GA = 27 (IQR = 27-28) weeks; md bodyweight (BW) = 1030 grams (IQR = 880-1200)). Among the 575 (26.6%) infants who died before discharge, 216 (37.6%) died following a decision of WIC. The proportion of WIC was the highest in the 24-26-week group (13.7%) instead of the 22-23-week group (6.3%); the highest proportion of WIC was in the 501-750-gram group (16.2%) and was at 14.3% in the 751-1000-gram group, while infants <500 g did not undergo WIC (**Table 1**). A lower percentage of artificially conceived infants (11.2% vs 20.9%, *P* = 0.001) and male infants (43.1% vs 50.1%, *P* = 0.038) underwent WIC. The maternal age was higher in the MIC (md = 32 (IQR = 29-36)) than the WIC group (md = 31 (IQR = 28-35)). The Apgar at five minutes and SNAPPE-II revealed no statistical significance between the two groups.

**Table 1:** Maternal and peripartum characteristics between two groups

Characteristics	WIC (n = 216), n (%)*	MIC (n = 2012), n (%)*	*P*-value
Gestational age in weeks (postnatal age in days)			<0.001
*22 (0) to 23 (6)*	1 (6.3)	15 (97.7)	
*24 (0) to 24 (6)*	9 (15.3)	50 (84.7)	
*25 (0) to 25 (6)*	20 (15.0)	113 (85.0)	
*26 (0) to 26 (6)*	45 (12.5)	305 (87.5)	
*27 (0) to 27 (6)*	65 (10.0)	583 (90.0)	
*28 (0) to 28 (6)*	76 (7.4)	946 (92.6)	
Birth weight in grams			<0.001
*<501*	0 (0)	23 (100.0)	
*501-750*	45 (19.5)	186(80.5)	
*751-1000*	106 (12.8)	722 (87.2)	
*1001-1250*	53 (6.6)	754 (93.4)	
*>1250*	12 (3.5)	327 (96.5)	
Maternal age, median (IQR)	31 (28,35)	32(29,36)	0.005
Outborn	52/216 (8.2)	184/2012 (8.2)	0.390
Gestational diabetes mellitus	28/216 (13.0)	297/2012 (14.8)	0.382
Pregnancy-induced hypertension	46/16 (21.3)	36/2012 (17.9)	0.225
Antenatal corticosteroids	129/163 (79.1)	1470/2012 (76.7)	0.234
Assisted conception	24/215 (11.2)	416/1986 (20.9)	0.001
Caesarean section	106/216 (49.1)	1052/2000 (52.6)	0.707
Multiple births	62/216 (28.7)	527/2012 (26.2)	0.426
Male	93/216 (43.1)	1008/2012 (50.1)	0.038
Intubation in delivery room	100/207 (48.3)	795/18 (42.6)	0.116
Apgar <7 at five minutes	48/210 (22.8)	600/1995 (30.1)	0.069
SNAPPE-II>20	111/212 (52.4)	1162/2003 (57.8)	0.083
Admission temperature <36°C	96/214 (44.9)	771/2009 (38.4)	0.081

WIC – withdrawal of care, MIC – maximal intensive care, IQR – interquartile range

*Denominators (i.e. total n) varied according to the number of missing data for each variable.

### Mortalities and major morbidities

The mortalities and major morbidities for each GA group are shown in **Table 2**. After excluding death after WIC, the mortality rates declined from 75.4% at 22-24 weeks to 31.8% at 25-26 weeks and 11.6% at 27-28 weeks. Survival rates without severe neurological injury were 6.7% for infants at 22-23 weeks, 28.0% at 24 weeks, 56.7% at 24 weeks, 61.7% at 25 weeks, 79.9% at 26 weeks, and 84.4% at 27 and 28 weeks. Complications rates were 10.0% for SNI, 20.9% for BPD, 6.0% for NEC, and 8.6% for ROP.

**Table 2:** Mortality and morbidities excluding WIC at each gestational age group*

Variable	22-23w (n = 15), n/N (%)	24w (n = 50), n/N (%)	25w (n = 113), n/N (%)	26w (n = 305), n/N (%)	27w (n = 583), n/N (%)	28w (n = 946), n/N (%)	Total (n = 2012), n/N (%)
**Mortality, n (%)**	14/15 (93.3)	35/50 (70.0)	41/113 (36.3)	92/305 (30.2)	82/583 (14.6)	95/946 (10.0)	359/2012 (17.8)
**Postnatal time until death in days**							
<1	5/14 (35.7)	15/35 (42.9)	15/41 (36.6)	17/92 (18.5)	15/82 (18.3)	21/95 (22.1)	88/359 (24.5)
1-7	5/14 (35.7)	11/35 (31.4)	10/49 (20.4)	33/92 (35.9)	33/82 (40.2)	33/95 (34.7)	125/359 (34.8)
8-28	4/14 (28.6)	7/35 (20.0)	12/49 (24.5)	25/92 (27.2)	27/82 (32.9)	25/95 (26.3)	100/359 (27.9)
>28	0/14 (0)	2/35 (5.7)	4/49 (8.2)	17/92 (18.5)	7/82 (8.5)	16/95 (16.8)	46/359 (12.8)
**Major morbidity**							
SNI	2/15 (13.3)	11/50 (22.0)	15/113 (13.3)	39/305 (12.8)	59/583 (10.1)	76/946 (8.0)	202/2012 (10.0)
BPD	0/1 (0)	11/25 (44.0)	36/87 (41.4)	85/236 (36.0)	111/508 (21.9)	114/845 (13.5)	357/1702 (20.9)
NEC	1/15 (6.7)	5/50 (10.0)	9/113 (8.0)	19/305 (6.2)	43/583 (7.4)	44/946 (4.7)	121/2012 (6.0)
ROP	1/3 (33.3)	4/24 (16.7)	13/87 (14.9)	25/249 (10.0)	33/526 (6.3)	76/879 (8.6)	152/1768 (8.6)
Survival to discharge without SNI	1/15 (6.7)	14/50 (28.0)	65/113 (57.5)	188/305 (61.6)	466/583 (79.9)	799/946 (84.4)	1533/2012 (76.2)

WIC – withdrawal of care, SNI – severe neurological injury, NEC – necrotic enterocolitis of newborn, BPD – bronchopulmonary dysplasia, ROP – retinopathy of prematurity, MIC – maximal intensive care, w – weeks

*The denominator for BPD was survivors at 36 weeks; the denominator for ROP was survivors who had an eye exam.

### Internal comparison between NICUs in SNN

**Figure 1:**
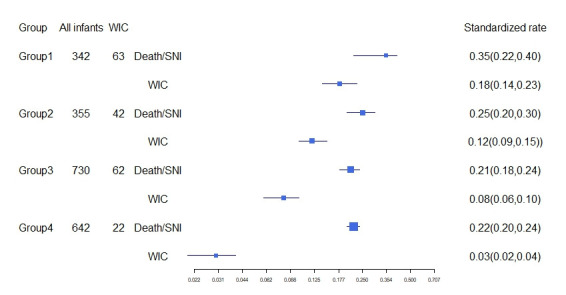
Internal comparison between NICUs in SNN. The grouping criteria were based on the quartiles of the proportion of MIC from highest to lowest into four groups. The incidence of mortality or SNI after MIC was calculated after standardization of potential confounders including male gender, antenatal corticosteroids, SNAPPE-II>20 and multiple births. The denominator for death or SNI was infants received MIC. SNI – severe neurological injury, WIC – withdrawal of care, MIC – maximal intensive care.

In the internal comparison, we divided NICUs into four groups per the proportion of WIC; the group with higher proportion of WIC also had a higher rate of death or SNI after MIC. While the proportion of WIC in groups 1 and 4 were 18% vs 3%, the rate of death or SNI were 35% vs 22% (**Figure 1**).

### Multivariable adjusted hazard ratios for death or SNI according to GA

**Figure 2:**
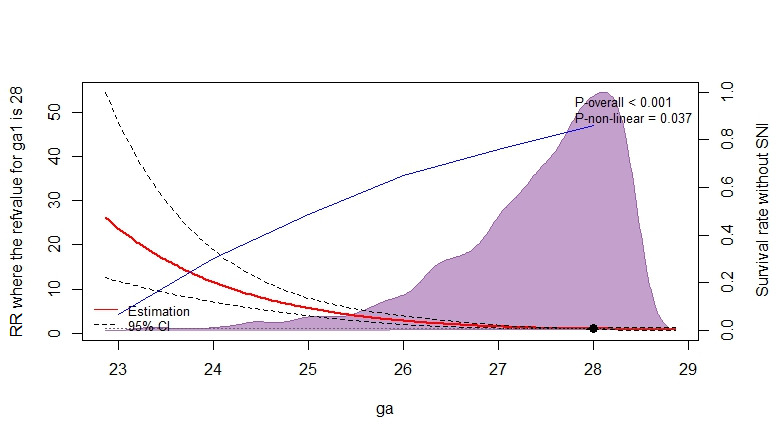
The restricted cubic spline curves based on multivariable adjusted hazard ratios for death or SNI according to GA. Solid red line shows the multivariable adjusted hazard ratios, with dashed black lines showing 95% CIs derived from restricted cubic spline regressions with three knots. The dark blue shows the survival rate without SNI according to GA. Purple curve shows the fraction of the population with different GA. We adjusted the analyses for male gender, antenatal corticosteroids, SNAPPE-II>20 and multiple births at baseline.

The risk of death or SNI gradually decreased with increasing GA (**Figure 2**). Compared with the threshold at 28 weeks, the relative risk for death or SNI were 1.53 (95% confidence interval (CI) = 1.26-1.86) at 27 weeks, 2.32 (95% CI = 1.73-3.11) at 26 weeks, 3.62 (95% CI = 2.43-5.40) at 25 weeks, 8.91 (95% CI = 4.69-16.96) at 24 weeks, and 29.8 (95% CI = 11.69-76.04) weeks.

## DISCUSSION

The results of our study, which covered 40 NICUs in Sino-China, indicated that viability after 25 weeks should not be underestimated compared to the traditional criteria of GA>28 weeks for live births. An active attitude toward saving lives might be an important factor in improving survival rates, and the resuscitation threshold should be gradually changed based on reliable capacity.

Knowledge of reliable outcomes is crucial for providing adequate prenatal management and informing medical decisions. Thus, we prospectively defined the mode of death, with each participating unit confirming the use of a consistent criterion. Due to socioeconomic reasons, we did not count in the denominator in our statistical analysis for WIC. Survival rates without SNI reached 57.5% for infants at 25 weeks and 61.6% for those at 26 weeks. However, mortality due to WIC accounted for nearly 40% of all deaths. Among all live births, deaths without active treatment (including palliative care in the delivery room and WIC after admission to NICU) accounted for 70.7% (n = 522/688) of all deaths. How small is not too small in present-day China, however, still warrants further exploration.

We found that a more aggressive prenatal preparation and resuscitation for preterm infants at 25-27 weeks before delivery, rather than a postnatal decision for further care, may benefit a larger absolute number of infants in China. Although our study is not based on a national cohort, its geographic area covers a quarter of China's population and is representative of those with similar levels of medical care. Countries or regions shoulds analyse baseline data to inform physicians to reduce death caused by medical and non-medical reasons.

The workable recommendations can be beneficial in improving outcomes for preterm infants. For example, the publication of the Swiss guidelines was followed by a significantly improved survival of infants born extremely preterm [25]. The option of intrauterine transfer, caesarean section, and application of antenatal corticosteroids and prenatal magnesium sulphate are regulated in guidelines [26,27]. The timing of deaths in the MIC group suggests that the most deaths occur within seven days, while this number was higher within 24 hours for infants under 26 weeks, which may be related to inadequate perinatal management. Paediatricians, obstetricians, and midwives must have information parity so perinatal management can be more standardised and adequate, and so that parents can obtain data on outcomes and confidence in prenatal care.

We found China has a similar mortality rate to networks in developed countries with relatively higher mortality rate, such as the Spanish Neonatal Network (23.4%) or the United Kingdom Neonatal Collaborative (15.8%), but not with others like the Finnish Medical Birth Register (9.6%) and the Neonatal Research Network of Japan (7.0%) [28]. However, compared to the last decade, great improvements in rescue capacity have also been achieved nowadays [10,11]. Studies have shown that aggressive treatment of younger GA groups also improves survival of older GA groups. In a comparison of attitudes towards treatment of infants with extreme survival, the units treated more aggressively also had better survival and long-term outcomes across GA groups [29-31]. In the comparison between different NICUs in SNN, units with a lower withdrawal rate also showed a lower mortality rate after MIC, and the incidence of severe neurological injury did not increase, suggesting that an active attitude toward care might be crucial in improving survival rates.

For industrialised countries, the resuscitation threshold constantly changed from the 1960s to 1970s; it included infants with GA>28 weeks and BW>700 g, but now, there is general agreement for comfort care at 22 weeks’ GA and active care at 25 weeks’ GA [32-34]. This change in thresholds is largely based on multi-centre studies of immediate and long-term outcomes, and the grey zone is more narrowly defined [34]. Except for the United States, the top 10 countries globally per the number of preterm births are all developing countries, yet few have guidelines for resuscitation [35]. Even those which do exhibit a very wide range of resuscitation thresholds, for example, the potential viability in Philippine refers to extremely preterm birth from 24-28 weeks’ gestation [36]. Moreover, more complex and interrelated socioeconomic and cultural reasons influenced specific treatment decisions [21,37]. In our study, we used death and SNI, the two most severe immediate outcomes with the greatest impact on preterm infants as the primary outcomes, we found it is feasible to change recommendations for active recovery. Description of outcomes and exploration of more appropriate recommendations could potentially be adapted by more countries and areas facing similar dilemma.

This study has several limitations. In China and other developing countries, where the neonatal collaborative network has only recently been established, data on long-term follow-ups are not yet available [38], thus the use of death or SNI to represent the most severe prognosis is also the most likely indicator of a poor outcome. Furthermore, while statistical result indicated that the risk ratio ranged from 1.53 to 3.62 at 27-25 weeks, we could only provide reference for clinic practice, and a larger sample size may provide more scientific reference. Additionally, there are disparities of the NICU at different levels of care, so our study does not accurately provide specific guidelines for each level of NICU.

## CONCLUSIONS

Descriptive data and statistical analyses demonstrated that the rescue limit should be adjusted from 28 to 25 weeks, which may help make appropriate decisions. Future studies must assess the impact of this change, including the long-term outcomes of infants, the societal and economic burden on families, and cost and services utilisation of the health care system.

## Additional material


Online Supplementary Document
