# Supplementary material for: Determining resuscitation threshold for extremely preterm infants based on the survival rates without severe neurological injury
Source: J Glob Health. 2023 May 26;13:04059. doi: 10.7189/jogh.13.04059 (PMC10210526; doi:10.7189/jogh.13.04059)
Supplement: Online Supplementary Document [file jogh-13-04059-s001.pdf]

SUPPLEMENT

Table S1. All delivery data at birth by Gestational Age

	No. (%) of Events						
Gestational Age, wk	All Infants (N =5838)	TOP† (n =690)	Stillbirths† (n =2569)	Live Births† (n =2579)	Deaths in delivery room after palliative care‡ (n =306)	Deaths in delivery room after active care‡ (n =45)	Infants admitted to NICUs (n =2228)‡
22^{+0}-23^{+6}	1058	221(20.9)	782(73.9)	55(5.2)	31(56.4)	8(14.5)	16(29.1)
24^{+0}-24^{+6}	787	104(13.2)	576(73.2)	107(13.6)	42(39.3)	6(5.6)	59(55.1)
25^{+0}-25^{+6}	810	140(17.3)	461(56.9)	209(25.8)	66(31.6)	10(4.7)	133(63.6)
26^{+0}-26^{+6}	963	91(9.4)	397(41.2)	475(49.3)	116(24.4)	9(1.9)	350(73.7)
27^{+0}-27^{+6}	997	73(7.3)	230(23.1)	694(69.6)	40(5.8)	6(0.9)	648(93.4)
28^{+0}-28^{+6}	1223	61(5.0)	123(10.1)	1039(85.0)	11(1.1)	6(0.6)	1022(98.4)

Abbreviations: NICU= neonatal intensive care unit; TOP= termination of pregnancy for maternal and fetal reasons (other than congenital anomalies).

† Related to all births.

‡Related to live births.

Table S2. NICU medical resources at different levels

NICU level	NICU	beds	Conventional ventilation	High frequency invasive mechanical ventilationon	Continuos Positive Airway Pressure	Surfatant	Pediatric Surgery Consultation and Surgery
Provincial	a	40	9	6	16	Y	Y
	b	45	8	6	17	Y	Y
	c	30	7	4	12	Y	Y
	d	44	10	7	11	Y	Y
Prefecture	e	30	6	2	11	Y	Y
	f	35	5	2	5	Y	Y
	g	10	3	1	6	Y	Y
	h	63	8	6	12	Y	Y
	i	34	7	4	8	Y	Y
	j	50	10	9	14	Y	N
	k	30	8	2	18	Y	Y
	l	40	6	2	12	Y	Y
	m	32	2	1	4	Y	N
	n	60	9	6	18	Y	Y
	o	37	11	5	12	Y	Y
	p	40	11	8	21	Y	Y
	q	20	5	4	8	Y	Y
	r	20	4	22	6	Y	Y
	s	60	12	6	30	Y	Y
	t	100	9	2	23	Y	Y
	u	27	5	2	10	Y	Y
	v	20	5	2	2	Y	N
	w	50	6	2	13	Y	Y
	x	30	3	2	7	Y	Y
	y	62	17	5	23	Y	Y
	z	20	4	1	12	Y	Y
	aa	44	9	4	16	Y	Y
	ab	35	7	4	15	Y	N
	ac	28	5	2	10	Y	Y
	ad	30	7	5	13	Y	Y
	ae	35	7	4	7	Y	Y
	a	45	8	3	12	Y	Y

	f						
	ag	20	4	1	6	Y	N
	ah	35	3	2	8	Y	N
County	ai	8	1	0	2		
	aj	15	5	2	5	Y	N
	ak	12	4	2	8	Y	Y
	al	10	3	1	6	Y	Y
	am	16	3	2	5	Y	Y
	an	10	3	2	4	Y	N
